# Radon Improves Clinical Response in an Animal Model of Rheumatoid Arthritis Accompanied by Increased Numbers of Peripheral Blood B Cells and Interleukin-5 Concentration

**DOI:** 10.3390/cells11040689

**Published:** 2022-02-16

**Authors:** Lisa Deloch, Stephanie Hehlgans, Michael Rückert, Andreas Maier, Annika Hinrichs, Ann-Sophie Flohr, Denise Eckert, Thomas Weissmann, Michaela Seeling, Falk Nimmerjahn, Rainer Fietkau, Franz Rödel, Claudia Fournier, Benjamin Frey, Udo S. Gaipl

**Affiliations:** 1 Department of Radiation Oncology, Universitätsklinikum Erlangen, Friedrich-Alexander-Universität Erlangen-Nürnberg, 91054 Erlangen, Germany; michael.rueckert@uk-erlangen.de (M.R.); ann-sophie.flohr@uk-erlangen.de (A.-S.F.); thomas.weissmann@uk-erlangen.de (T.W.); rainer.fietkau@uk-erlangen.de (R.F.); benjamin.frey@uk-erlangen.de (B.F.); udo.gaipl@uk-erlangen.de (U.S.G.); 2 Translational Radiobiology, Department of Radiation Oncology, Universitätsklinikum Erlangen, Friedrich-Alexander-Universität Erlangen-Nürnberg, 91054 Erlangen, Germany; 3 Department of Radiotherapy and Oncology, University Hospital Frankfurt, Theodor-Stern-Kai 7, 60590 Frankfurt am Main, Germany; stephanie.hehlgans@kgu.de (S.H.); franz.roedel@kgu.de (F.R.); 4 GSI Helmholtzzentrum für Schwerionenforschung, 64291 Darmstadt, Germany; a.maier@gsi.de (A.M.); a.hinrichs@gsi.de (A.H.); d.eckert@gsi.de (D.E.); c.fournier@gsi.de (C.F.); 5 Department of Physics, Goethe Universität Frankfurt am Main, 60323 Frankfurt am Main, Germany; 6 Department of Biology, Institute of Genetics, Friedrich-Alexander-Universität Erlangen-Nürnberg, 91058 Erlangen, Germany; michaela.seeling@fau.de (M.S.); falk.nimmerjahn@fau.de (F.N.)

**Keywords:** inflammation, radon therapy, immune system, macrophages, serum transfer arthritis, monocytes, T cells, B cells, rheumatoid arthritis, anti-oxidative system

## Abstract

Radon treatment is used as an established therapy option in chronic painful inflammatory diseases. While analgesic effects are well described, little is known about the underlying molecular effects. Among the suspected mechanisms are modulations of the anti-oxidative and the immune system. Therefore, we aimed for the first time to examine the beneficial effects of radon exposure on clinical outcome as well as the underlying mechanisms by utilizing a holistic approach in a controlled environment of a radon chamber with an animal model: K/BxN serum-induced arthritic mice as well as isolated cells were exposed to sham or radon irradiation. The effects on the anti-oxidative and the immune system were analyzed by flow-cytometry, qPCR or ELISA. We found a significantly improved clinical disease progression score in the mice, alongside significant increase of peripheral blood B cells and IL-5. No significant alterations were visible in the anti-oxidative system or regarding cell death. We conclude that neither cell death nor anti-oxidative systems are responsible for the beneficial effects of radon exposure in our preclinical model. Rather, radon slightly affects the immune system. However, more research is still needed in order to fully understand radon-mediated effects and to carry out reasonable risk-benefit considerations.

## 1. Introduction

The noble gas radon is a naturally occurring radioactive substance. While it greatly contributes to the exposure from naturally occurring sources of ionizing radiation (IR) it is also widely used in the treatment of benign inflammatory and non-inflammatory diseases, in many countries. Today, it is widely accepted that the emitting alpha-particles from radon in these springs are responsible for the main dose and thus for several beneficial effects [1,2,3]. At present, the analgesic effects of radon therapies are exploited in the form of bathing in water containing radon, drinking radon-infused water or via inhalation therapy in galleries or caves with enhanced radon concentrations [2,3,4,5]. 

There are many indications for which radon therapy can be utilized: among them are rheumatoid arthritis (RA), ankylosing spondylitis (AS), psoriasis arthritis, osteoarthritis (OA) and others. Many of the patients suffering from these diseases have undergone prior therapies with limited success. Next to the discomfort of patients that fail conventional therapies, all of these diseases bear a relatively high socioeconomic burden. Here, a therapy utilizing radon might be a useful additional therapy option, especially with regard to pain management [1,5,6,7,8], similar to observations made in the treatment of chronic, inflammatory diseases with low doses of X-rays [9]. 

Various studies have shown that patients that have undergone radon therapy benefit from long-lasting pain-reducing effects [1,2,6,7,8,10,11,12] as reviewed by Maier et al. and Falkenbach et al. [2,10]. Next to the known analgesic effects, some hints on underlying molecular mechanisms have already been shown in pre-clinical models and patient studies. Among the most prominent findings in pre-clinical studies were discoveries concerning alterations of the anti-oxidative system with special emphasize on superoxide dismutase (SOD)1 as a key molecule [13,14,15,16,17,18] and in the immune system. For the latter, Nagarkatti et al. reported an alteration of T cell numbers after radon exposure in lymphoid organs of mice as well as alterations in the numbers of lymphocytes and macrophages [19]. In patient studies, Cucu et al. reported alterations after radon spa treatment in the frame of the explorative RAD-ON01 study, that were related to bone metabolism, such as a decrease in collagen fragments in patient serum alongside a shift in T cell subpopulations [20]. In the same cohort Rühle et al. found modulations of immune cells in the peripheral blood, including alterations of neutrophils, eosinophils and dendritic cells (DCs) as well as long lasting effects in monocyte and T cell numbers and a temporary very slight increase of B cells. However, the most prominent effects were not visible in total cell numbers, but rather in the activation status of the examined cells [3]. Kullmann et al. further reported a significant increase in TGFβ, which similar to alterations they found in interleukin (IL)-18 release, had a positive correlation with pain perception in the examined patient cohort [21].

One of the suggested potential mechanisms behind the effects of radon therapy, is the anti-oxidative system [13,14,15,16,17,18], as it has also been reported after treatment with low-doses of X-rays [22]: reactive oxygen species (ROS) are essential signaling molecules in a variety of cellular processes [23,24]. Oxidative stress, however, resulting from an excessive ROS or oxidant amount overcharging the cellular capability to mount a sufficient anti-oxidant response, is harmful for the cells, causes macromolecular damage and can lead to diseases such as diabetes, atherosclerosis or cancer [24]. ROS can arise from intrinsic processes, but it can also be induced by external influences such as drug exposure and IR [25]. To scavenge excessive amounts of ROS, cells have developed anti-oxidant defense mechanisms, including anti-oxidative enzymes. For example, SOD dismutates superoxide anions in hydrogen peroxide and this, in turn, is converted into water and oxygen by, e.g., glutathione peroxidase (GPx) and catalase [26]. An important sensor of oxidative stress is the transcription factor nuclear factor-erythroid-2-related factor 2 (Nrf2), which is restored in the cytoplasm through binding of an inhibitor, which mediates rapid ubiquitination and degradation of Nrf2. Upon oxidative stress, Nrf2 is released from Keap1 and induces an anti-oxidant cellular defense to restore the redox balance [25]. 

One has to be aware that in all of these studies, treatment was carried out differently (e.g., via inhalation, uptake over the skin). Further, it was not possible due to the nature of the therapy to exclude or synchronize other factors such as elevated temperature, humidity or spa effect that might have contributed to the observed results, and lastly, activity concentrations and thus applied doses varied considerably (radon baths 0.8–3 kBq/L radon and in radon caves or galleries, concentration in the air ranged from 37–160 kBq/m^3^, depending on location) as summed up by Falkenbach et al. [10].

Next to these beneficial properties of radon treatment, there are also concerns with regard to potential side effects and risk factors, especially for the development of cancer. However, to date, no significant results, even for larger patient cohorts are established. This is mainly due to the very low doses that are used in radon treatments which also lead to relatively small measurable effects that are often masked by other lifestyle factors (e.g., smoking) [27]. In that matter, Paz et al. [4] performed analyses in patients that were exposed to radon treatment and compared them to healthy donors. They found that radon treatment had no significant impact on aberration frequency or the fraction of complex aberrations in human peripheral blood lymphocytes. They thus conclude that in their cohort and based on biological dosimetry utilizing aberration analyses, no increased health risk was detected [4].

Nevertheless, a careful risk-benefit assessment should always be carried out prior to treatment with IR. Therefore, we aimed to take a closer look on the molecular mechanisms under controlled conditions in an in vivo and ex vivo setting taking multiple parameters such as clinical progression, the involvement if the immune system alongside systemic cytokine alterations as well as examinations of the anti-oxidative system into account.

One disease that is treated with radon and that also has been linked to oxidative stress [28] is RA. We therefore chose the K/BxN serum-transfer arthritis model as our model system for this study, as it closely resembles antibody-mediated RA in patients. As it is an antibody-mediated model, immune cells, as well as cytokines and chemokines and factors involved in pain perception are all involved in this model. This leads to a progression of the disease also including bone erosions and pannus formation. Further, as serum of K/BxN mice can be collected and then injected in other strains [29], obtaining larger groups of sex- and age-matched animals alongside similar onset and progression of RA is another advantage, especially in radon research, as doses are low and effects are expected to be small. 

As mentioned above, further challenges when studying radon-induced effects include the uniformity of applied doses over all treatment groups, the measurements of actual radon uptake and the feasibility of placebo controlled, randomized studies. Similarly, most other in vitro studies mainly use α-sources to carry out their experiments in order to have a better controlled environment, but which neglect the effects of mixed radiation qualities of radon decay. Therefore, we decided to opt for a radon chamber that was built at the *GSI Helmholtzzentrum für Schwerionenforschung* (GSI), Darmstadt [27] where parameters are easier to control than in actual radon galleries. Here, we were also able to focus on radon-induced effects, as other parameters such as elevated temperature or humidity are able to be set, controlled, or switched off, as needed. This allowed us to carry out in vivo and ex vivo analysis using radon in a controlled environment.

## 2. Materials and Methods

### 2.1. Animal Upkeep and Clinical Evaluation

**Figure 1:**
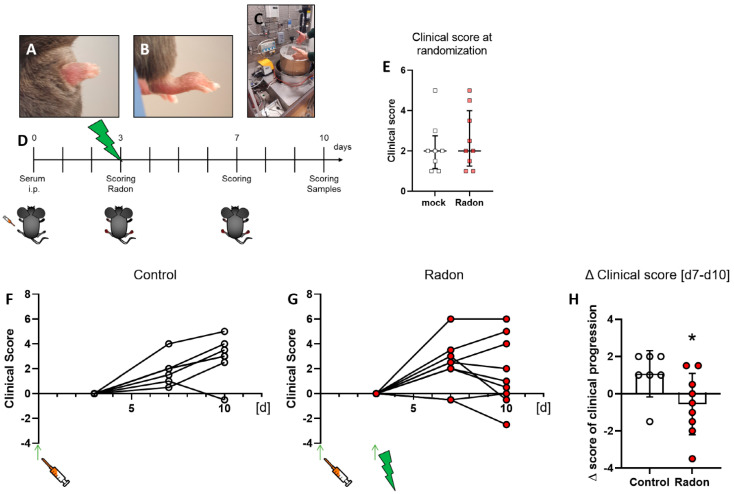
Exposure to radon gas for one hour results in an improved clinical score in K/BxN serum-induced C57Bl76 mice in comparison to mock-treated controls. Female C57Bl/6 mice were injected i.p. with 200 µL or 300 µL, depending on the respective serum batch, of pooled K/BxN serum. Within a few days, mice develop a visible swelling of the paws (**A**,**B**). On d3 after the injection, mice were scored and randomly distributed into two groups (**E**) and placed into the radon chamber (**C**) for treatment. While one group received mock treatment, the other group was exposed to radon gas. Both groups were scored again on d7 and d10. On d10 mice were sacrificed and samples (whole blood, serum, bone marrow and hind feet) were collected for further analysis ((**D**), timeline). Clinical score of serum-induced mice was determined according to swelling (0—no swelling, 5—complete swelling of paws and toes), whereas the score of both hindlegs/mouse was added up for final evaluation. 6 out of 9 animals in the radon group (**F**) showed an improvement in clinical score whereas only one in 8 control animals (**G**) showed improvement during the time of observation. These effects are also visualized in (**H**), where the Δ clinical score [d7–d10] of clinical progression is significantly better in radon treated animals when compared to mock controls. Figure shows data of two independent experiments with in total 10 mice/group and is shown as Median + IQR. Animals that did not show swelling on d3 were removed from the experiment; n_control_ = 7; n_Radon_ = 9. Statistics were calculated using Mann-Whitney-U test; (* *p* < 0.05).

10 weeks old female C57Bl/6 mice were ordered from Janvier Labs (Le Genest-Saint-Isle, France) and maintained in the animal facility at GSI. All animal procedures have been approved by the “*Regierung von Unterfranken*” as well as the “*Regierung von Hessen*” (approval numbers: 55.2-DMS-2532-2-114 from 13 November 2015 and 10 December 2015; TS-3/14 from 21 February 2014) and were conducted in accordance with the guidelines of Federation of European Laboratory Animal Science Associations (FELASA). Pooled serum from transgenic K/BxN mice was provided by the group of Falk Nimmerjahn. 200 µL or 300 µL serum, depending on the determined efficacy of harvested sera, was injected intraperitoneally into the mice, Figure 1A,B. This model was first reported by Kouskoff et al. [30] and was discovered by crossing T cell receptor (TCR) transgenic KRN mice with autoimmune-prone, non-obese diabetic (NOD) mice. The F1 generation of these mice was called K/BxN and developed severe arthritis within the first couple of weeks. Serum of these mice resulted in a reproducible arthritis in many mouse strains when being transferred to them [29]. 

Arthritis score evaluation was carried out in a blinded manner with a score system ranging from 0 (no swelling) to 3 (massive swelling) as described previously [31]. The experimental set-up for in vivo radon exposure can be seen in Figure 1C,D.

### 2.2. Cell Culture Experiments

Bone marrow (BM) from 6-week-old, female C57Bl/6 mice (Janvier Labs) was isolated from the long bones of the hind legs (femur, tibia), followed by lysis of erythrocytes for 5 min at room temperature. Next, cells were frozen in 10%DMSO/FCS at −80 °C and transported to the GSI on dry ice. Prior to experiments, cells were defrosted and seeded into 6 Well plates in DMEM (Gibco Life Technologies, Carlsbad, CA, USA) supplemented with 10%FCS (Sigma Aldrich, St. Louis, MO, USA) and 1% Penicillin Streptomycin (Gibco Life Technologies). Monocyte and macrophage medium were further supplemented with 5 ng mL M-CSF (Peprotech, Rocky Hill, NJ, USA). BM was seeded 1 h prior to treatment. For monocytes, BM cells were seeded in 10 cm dishes 6 h prior to mock or radon treatment and the non-adhering fraction was transferred into 6 Well plates 1 h before irradiation. For macrophage differentiation, BM cells were also seeded in 10 cm dishes for 6 h, the non-adhering fraction was then differentiated into M0 macrophages for 7 days in the presence of 5 ng/mL M-CSF. All cells were kept under standard cultivation conditions (37 °C, 5% CO_2_, 90% humidity).

### 2.3. Radon Treatment

Animals and cells were exposed to radon gas in a radon chamber in a controlled environment at GSI, Darmstadt, Germany for one hour as described in [27,32]. Mice were placed in a cage within the chamber, see Figure 1C, and exposed to radon gas for one hour, followed by fresh air for 30 min. 7 days after mock or radon exposure, animals were sacrificed and bone marrow, full blood, serum and organs were harvested for further analysis. Exposition conditions for in vivo experiments can be found in Table 1. 

Cells were exposed for 1 h and chamber was flushed with fresh air for 30 min afterwards. The optimum irradiation practice was tested out beforehand. Covering cells with 750 µL medium during treatment ensured that cells did not fall dry during this period, while radon treatment was carried out as effective as possible with the smallest possible liquid barrier. 24 h after the mock or radon treatment cells and supernatants (SN) were harvested and used for further experiments. Mean exposition parameters for ex vivo exposure are displayed in Table 2. 

### 2.4. Multicolor Flow Cytometry Analysis

**Figure A1:**
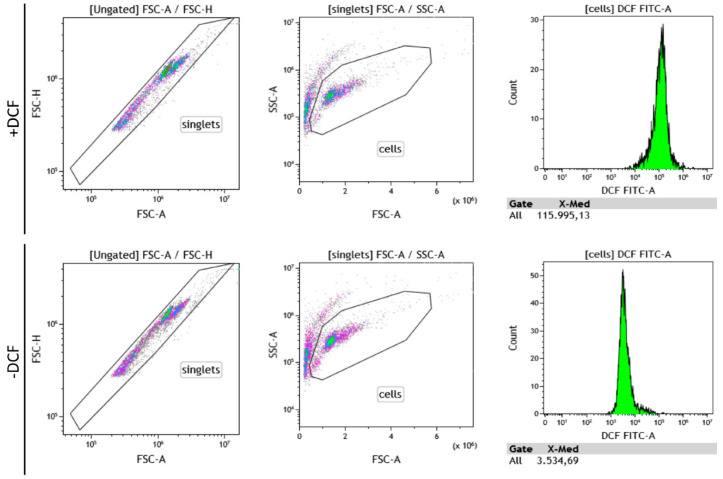
Exemplary gating strategy for 2′,7′-Dichlordihydrofluorescein-diacetat (DCF) measurements. For each condition, two wells were prepared, treated and washed. One well was subsequently treated with 2 µM DCF (+DCF), while the other one received 0 µM DCF (−DCF), followed by a 90 min incubation period at standard culture conditions in the dark. Afterward, cells were detached and DCF activity was measured. For analysis, ∆MFI was calculated from MFI(+DCF)–MFI(−DCF).

**Figure A2:**
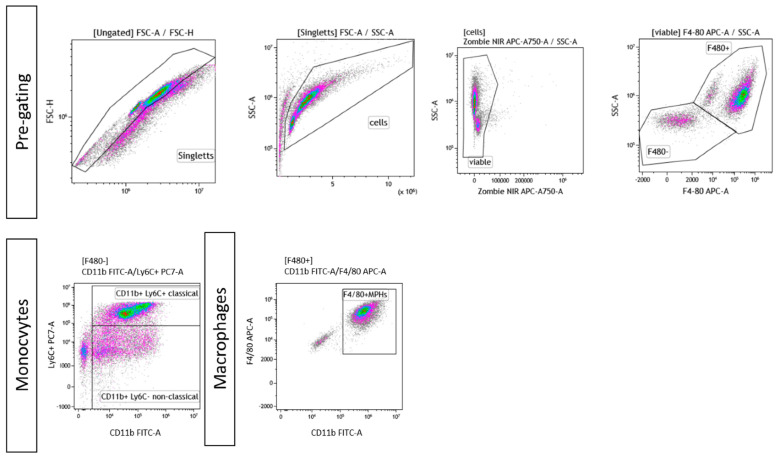
Exemplary gating strategy for ex vivo differentiated monocytes and macrophages. In both cases, singlets were pre-chosen, followed by gating of viable cells and a live/dead control using Zombie NIR. Afterward, cells were gated for F4/80+ or F4/80−. In case of monocytes, F4/80− cells were further classified into “classical” (F4/80-CD11b+Ly6C+) or “non-classical” (F4/80-CD11b+Ly6C−) monocytes. In case of ex vivo differentiated macrophages, F4/80+CD11b+ cells were considered to be macrophages.

For flow cytometry analysis whole blood and bone marrow (BM) of animals was collected. Erythrocytes in 100 µL whole blood were lysed by 0.12% formic acid for 10 s, followed by a neutralization step and subsequent fixation in 4% paraformaldehyde. For analysis of BM, 1 × 10^5^ cells/stain and for blood samples 35 µL of whole blood/stain was used. For ex vivo experiments, cells were collected 24 h after radon or mock exposure. Cells (lysed blood cells, BM as well as monocytes and macrophages) were resuspended in 50 µL Fc-block solution (eBioscience, San Diego, CA, USA) and incubated for 10 min at room temperature. Staining for antibody panels was then carried out at 4 °C for 30 min as described previously [33] and as it can be seen in Table A1 in Appendix A. For 2′,7′-Dichlordihydrofluorescein-diacetat (DCF) Assay, cells were washed and treated with either 2 µM DCF or 0µM DCF (Invitrogen, Waltham, MA, USA) for 90 min at standard culture conditions in the dark. Subsequently, cells were detached and analyzed in a flow cytometer. For evaluation, ∆mean fluorescence intensity (MFI) of (2 µM DCF)–(0 µM DCF) was calculated. Cells were analyzed using a CytoFLEX S flow cytometer and data was analyzed with the help of the Kaluza analysis software (both: Beckman Coulter, Brea, CA, USA). Gating strategies are depicted in Appendix A Figure A1 and Figure A2.

### 2.5. MSD^®^ Multi-Spot Assay System and Enzyme-Linked Immunosorbent Assays

Multiplex ELISA was carried out using the Meso Scale Discovery^®^ (MSD^®^; Rockville, MD, USA) system that was used according to the manufacturer’s recommendation. For in vivo experiments, the Proinflammatory Panel 1 (mouse) Kit V-Plex^®^ Assay as well as the TH17 Panel 1 (mouse) Kit V-Plex^®^ Assay were used. Samples were diluted according to the manufacturer’s recommendations, 1:2 for the Proinflammatory and 1:4 for the TH17 Panel, respectively. Subsequent analysis was carried out using MSD^®^ Discovery Workbench^®^. 

### 2.6. RNA Isolation and cDNA Synthesis

Cells were harvested 24 h after Radon exposure by centrifugation, washed once with PBS and the cell pellets were lysed in 300 µL lysis buffer ML (Macherey-Nagel, Düren, Germany) and stored at −80 °C. RNA was subsequently prepared with the NucleoSpin miRNA Kit (Macherey-Nagel, Düren, Germany) and 500 µg RNA was reverse transcribed using 200 Units M-MLV reverse transcriptase (Promega, Walldorf, Germany), dNTPs (500 µM; Carl Roth, Karlsruhe, Germany) and random hexamer primers (5 µM; Thermo Fisher Scientific, Darmstadt, Germany) for 10 min at 25 °C, 30 min at 37 °C and 30 min at 42 °C.

### 2.7. Quantitative Real-Time PCR (qPCR)

Expression of murine anti-oxidative enzymes Superoxide dismutase 1 (SOD1), Glutathione peroxidase 1 (GPx1), Catalase and the transcription factor Nuclear factor-erythroid-2-related factor 2 (Nrf2; gene name: nuclear factor, erythroid derived 2, like 2, Nfe2l2) was measured by quantitative real-time PCR (qPCR). Analyses were performed using a QuantStudio 5 Real-Time PCR System (Thermo Fisher Scientific), specific primers (0.5 µM), internal fluorescent TaqMan probes (0.25 µM; Eurofins Genomics, Ebersberg, Germany) and the GoTaq Probe qPCR Master Mix (Promega). The sequences of primers and probes are listed in Table A2. The following conditions were applied: 95 °C for 2 min and 40 cycles of 95 °C for 15 s and 60 °C for 1 min. Amplifications were performed using the standard curve methodology with serial dilutions of a pCR2.1 plasmid containing the specific fragment of the gene of interest, amplified with the individual forward and reverse primers (Table A2) and cloned using the Original TA Cloning Kit (Thermo Fisher Scientific). Plasmid DNA was purified with the NucleoBond Xtra Midi Plus EF Kit (Macherey-Nagel) according to the manufacturer’s recommendations and the correct insertion of the specific fragment was confirmed by sequencing (Eurofins Genomics). All samples were run in duplicate and normalized to the expression of the housekeeping gene hypoxanthine guanine phosphoribosyl transferase (HPRT).

### 2.8. Statistics

Data is presented as Median + interquartile range (IQR). Samples were tested for normal distribution and variance equality and subsequently analyzed using two-tailed Mann-Whitney-U test in comparison to untreated controls. Generation of graphs as well as statistical analysis was done using GraphPad Prism Software (Version 8.3.0; GraphPad software, Inc., San Diego, CA, USA). *p*-values ≤ 0.05 were considered to be statistically significant.

## 3. Results

### 3.1. Exposure to Radon Gas Significantly Improves Clinical Score in K/BxN Serum-Induced C57Bl76 Mice

As there is a lack in placebo-controlled studies for the treatment of musculoskeletal diseases with a radon inhalation therapy as it takes place in radon spa galleries, we exposed K/BxN serum-induced C57Bl76 mice to a radon inhalation therapy or mock treatment inside the radon chamber built at GSI, Darmstadt [27]. The mice remained in the cage within the radon chamber for one hour at room temperature (23 °C) and either received mock treatment or where exposed to a radon activity concentration of ~470 kBq/m^3^. In one of the most well-known radon spa galleries in Bad Gastein, Austria, patients receive approximately 44 kBq/m^3^ radon/therapy session in the course of 10 treatment sessions, each lasting for the duration of one hour. Unlike in our experiment where we wanted to focus on only radon-induced effects, therapy includes enhanced temperature (37–41.5 °C) and humidity (70–100%) [1,8]. 

Clinical score of mice was assessed as described in [31] at multiple time points throughout the experiments (see Figure 1D) which was also used to randomly distribute them into two groups at the beginning of the experiments (Figure 1E).When comparing clinical scores of mock treated animals with those that received radon therapy that was set in relation to the clinical score at the time of mock or radon treatment, we found that one in 7 mice from the mock group (Figure 1F) showed an improvement in clinical parameters while 7 in 9 mice from the radon group (Figure 1G) showed an improvement or remained stable in clinical score after radon treatment. When comparing d7 with d10 of the experiment, we found that radon had a significant impact on clinical progression of serum-induced mice (*p* = 0.0360, Figure 1H). 

### 3.2. The Anti-Oxidative System Has No Influence on the Clinical Response of K/BxN Serum-Induced Mice after Radon Therapy

**Figure 2:**
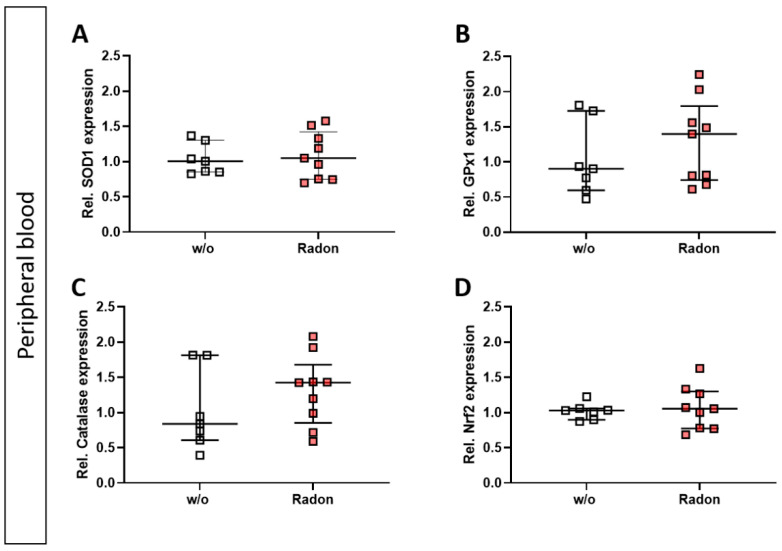
Exposure to radon gas for one hour results in a slightly increased expression of anti-oxidative enzymes Glutathione Peroxidase (GPx1) and Catalase in the peripheral blood of K/BxN serum-induced C57Bl76 mice in comparison to mock-treated controls. Serum-induced mice were either exposed to radon or were mock-treated (w/o) on day 3. After 7 days (day 10), mice were sacrificed and peripheral blood was collected and subjected to RNA isolation and quantitative real-time PCR to measure the expression of *Superoxide Dismutase 1* (*SOD1*) (**A**), *Glutathione Peroxidase 1* (*GPx1*) (**B**), *Catalase* (**C**) and *Nuclear factor-erythroid-2-related factor 2* (*Nrf2*) (**D**). Data are presented as Median + IQR and were derived from 2 independent experiments n_control_ = 7; n_Radon_ = 9. Statistics were calculated using Mann-Whitney-U test.

As mentioned above, ROS can be induced by extrinsic stimuli such as ionizing radiation. Therefore, we first evaluated a putative effect of radon exposure on the expression of anti-oxidative enzymes *Superoxide Dismutase 1* (*SOD1*), *Glutathione Peroxidase 1* (*GPx1*) and *Catalase* and their redox-sensitive transcription factor *Nuclear factor-erythroid-2-related factor 2* (*Nrf2*) in the peripheral blood of serum-induced mice. While the expression of *SOD1* and *Nrf2* was not modulated by radon gas inhalation, *GPx1* and *Catalase* expression was slightly increased in the radon group when compared to mock-treated animals (Figure 2A–D). 

### 3.3. Radon Treatment Alters Immune Cell Subsets in the Peripheral Blood but Not in the Bone Marrow

As suggested by Rühle et al. [3], we checked for modulations of immune cell subsets in the peripheral blood, as well as in the bone marrow, where, as described by Maier et al. [2], an elevated dose can be expected.

**Figure 3:**
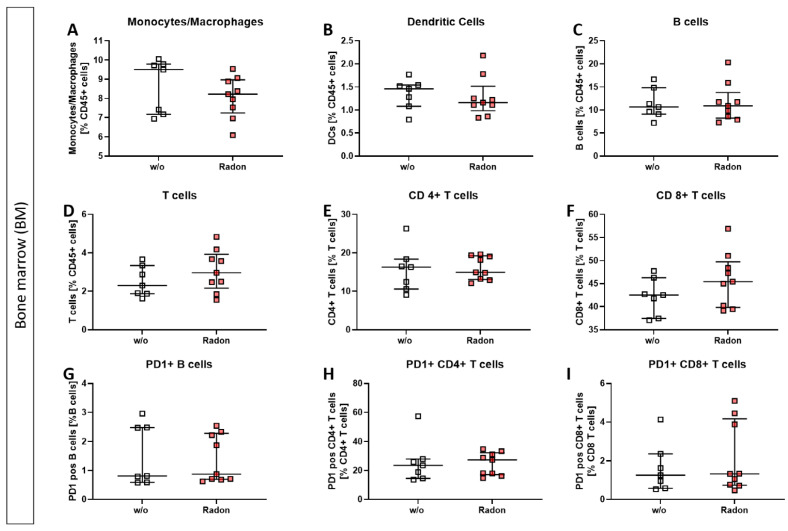
Inhalation of radon gas results only in minor immune cell alterations of the bone marrow in exposed mice. Bone marrow of the long bones of K/BxN serum-induced age and sex matched C57Bl/6 mice was collected, followed by lysis of erythrocytes and multicolor flow cytometry analysis for immune cell subtypes was carried out (**A**–**I**). Depicted are various immune cell subsets of radon and mock treated mice. Data shows two independent experiments with in total n_control_ = 7; n_Radon_ = 9 mice/group and is presented as Median + IQR. Statistics were calculated using Mann-Whitney-U test.

In the bone marrow of exposed mice, we found only mild alterations in immune cell subsets, such as a reduction in monocytes/macrophages, see Figure 3. 

**Figure 4:**
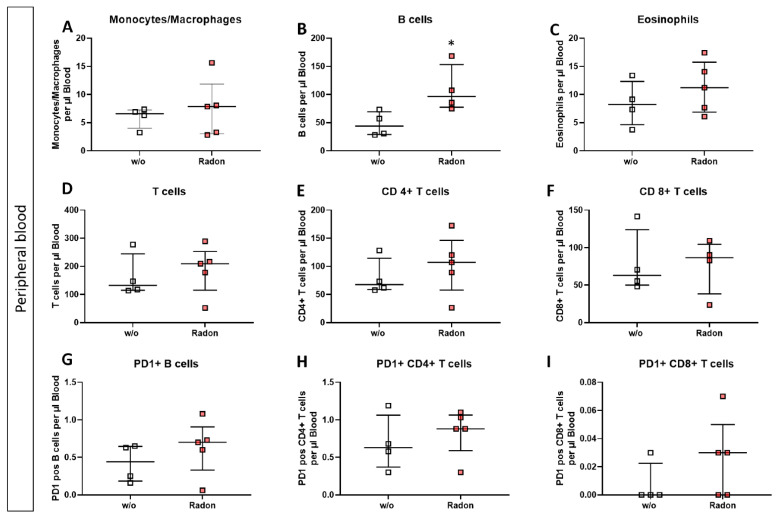
Inhalation of radon gas results in immune cell alterations in the peripheral blood of radon treated mice. Peripheral blood of K/BxN serum-induced age and sex matched C57Bl/6 mice was collected, followed by lysis of erythrocytes and subsequent multicolor flow cytometry analysis for immune cell subtypes was carried out (**A**–**I**). Depicted are various immune cell subsets of radon and mock treated mice. Data shows one independent experiment with in total n_control_ = 4; n_Radon_ = 5 mice/group and is presented as Median + IQR. Statistics were calculated using Mann-Whitney-U test (* *p* < 0.05).

In the peripheral blood, a significant increase of B cells in the radon group (Figure 4B, *p* = 0.0286) as well as a very slight increase in T cell subsets was evident. Likewise, PD1 was also slightly increased on B cells and CD8+ T cells after radon exposure (Figure 4D,E,G,I).

### 3.4. Radon Significantly Increases IL-5 Expression in the Serum of K/BxN Serum-Induced Mice

As alterations in the peripheral blood but not in the bone marrow were observed, we checked for cytokine expression using a multiplex ELISA system in the serum of radon and mock treated mice.

**Figure 5:**
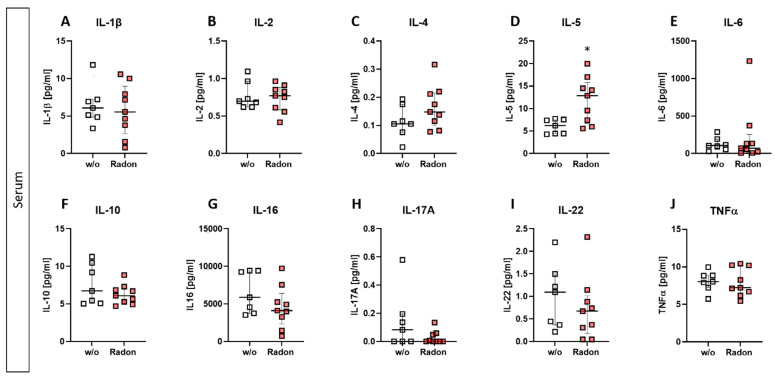
Exposure to radon gas for one hour results in altered cytokine expression levels in the serum of treated animals in comparison to mock treated animals. Serum of K/BxN serum-induced age and sex matched C57Bl/6 mice was collected and analyzed via MSD Multiplex Assay (**A**–**J**). Data shows cytokine levels of two independent experiments with in total n_control_ = 7; n_Radon_ = 9 mice/group and is presented as Median + IQR. Statistics were calculated using Mann-Whitney-U test (* *p* < 0.05).

While slight modulations were detected in some of the investigated cytokines (slight reduction of IL-16, IL-17A, and IL-22; minor increase in IL-4 (Figure 5C,G–I)), we found a significant increase of IL-5 within the radon group (Figure 5D, *p* = 0.0229). All other cytokines revealed no alterations after the radon treatment when being compared to the control group.

### 3.5. Radon Does Not Enhance Cell Death in Isolated Bone Marrow or Ex Vivo Differentiated Monocytes and Macrophages

As it is often suggested that ionizing radiation (IR) exerts some of its properties through the induction of cell death, we performed AnnexinV/Propidiumiodide (AxV/PI) staining in isolated bone marrow cells as well as in bone marrow-derived ex vivo differentiated monocytes and macrophages. 

**Figure 6:**
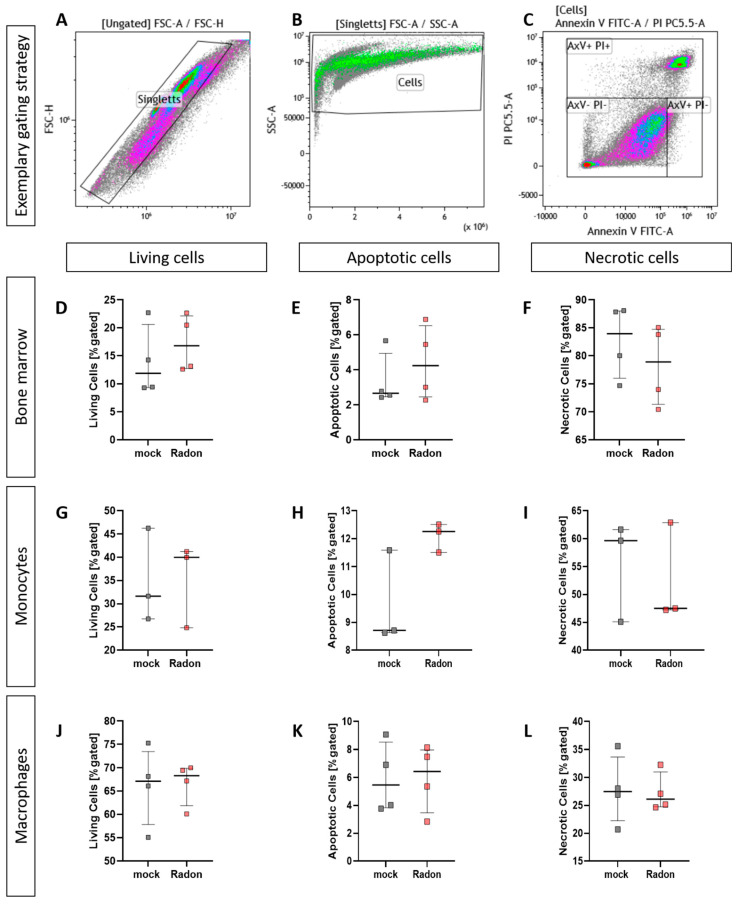
Ex vivo treatment of bone marrow cells as well as monocytes and macrophages with radon does not enhance cell death. Isolated bone marrow cells (168 kBq/m^3^) from C57Bl/6 mice as well as bone marrow-derived monocytes and macrophages (177 kBq/m^3^) were exposed to radon or mock treatment for one hour under standard cell culture conditions. 24 h after treatment, cells were collected, stained for AxV/PI and subsequently analyzed (**A**–**C**). For bone marrow cells (**D**–**F**), 4 cell pools, each derived from 10 C57Bl/6 mice, were exposed in two independently performed experiments. For monocytes (**G**–**I**) and macrophages (**J**–**L**), 3 and 4 cell pools were exposed in 3 independently carried out experiments. Data is presented as Median + IQR. Statistics were calculated using Mann-Whitney-U test.

When analyising cell death utilizing AxV/PI staining we found no significant influence of radon treatment on cell death in all ex vivo experiments we have performed (Figure 6).

### 3.6. Activation of Macrophages Increases the Expression of Anti-Oxidative Factors and Decreases ROS while Radon Exposure Only Moderately Changes the Anti-Oxidative System of Ex Vivo Cultivated Immune Cells

As monocytes and macrophages are considered to be key players in ROS-mediated effects, and as slight increase in *GPx1* and *Catalase* expression were found in the in vivo experiments (Figure 2), we checked for ROS release and anti-oxidative enzymes in an ex vivo setting. As it is also known, that inflammatory processes are often vital for low doses of IR [34,35], as it is also true in radon treatment, we added tumor necrosis factor (TNF)α to the cells in order to mimic inflammation.

**Figure 7:**
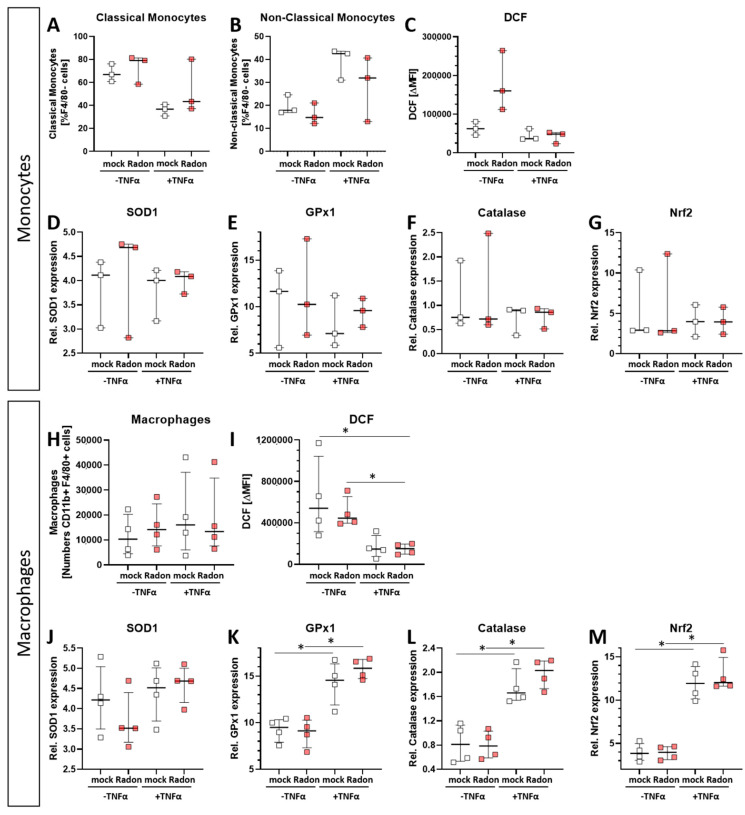
Ex vivo radon treatment of bone-marrow derived monocytes and macrophages has no effects on the anti-oxidative system while stimulation with TNF-α induces significant effects in macrophages. C57Bl/6 bone marrow-derived monocytes and macrophages were exposed to radon (177 kBq/m^3^) or mock treatment for one hour under standard cell culture conditions. 24 h after radon treatment, cells were collected, and either treated with DCF solution (**C**,**I**), stained with antibodies for multicolor flow cytometry analysis (**A**,**B**,**H**), or were collected for subsequent RNA analysis (**D**–**G**,**J**–**M**). Monocytes (**A**–**G**) and macrophages (**H**–**M**), 3 and 4 cell pools, respectively, were exposed in 3 independently carried out experiments. Data is presented as Median + IQR. Statistics were calculated using Mann-Whitney-U test (* *p* < 0.05).

Intracellular ROS levels, as measured via DCF Assay, in radon treated monocytes (Figure 7C) showed no significant alterations compared to mock treated ones. However, we observed significantly increased ROS levels (Figure 7I, *p* = 0.0286) in radon −TNFα treated samples compared to those with radon +TNFα treated ones as well as in mock treated ones compared to radon +TNFα treated ones (Figure 7I, *p* = 0.0286). This suggests a stronger effect of inflammatory stimulus than radon induced effects. We also found no differences in the distribution of classical or non-classical monocytes (Figure 7A,B) or macrophage numbers (Figure 7H). 

In order to examine the impact of pro-inflammatory stimulation with TNFα and radon exposure on the anti-oxidative system of immune cells ex vivo, we measured the mRNA expression of anti-oxidative enzymes *Superoxide Dismutase 1* (*SOD1*), *Glutathione Peroxidase 1* (*GPx1*) and *Catalase*, as well as their redox-sensitive transcription factor *Nuclear factor-erythroid-2-related factor 2* (*Nrf2*) in TNFα-stimulated vs. non-stimulated, mock-treated or radon-exposed monocytes and macrophages and mock vs. radon-exposed murine bone marrow. In monocytes, the expression of *SOD1*, *GPx1*, *Catalase* or *Nrf2* was not modulated by TNFα or Radon treatment (Figure 7D–G). 

By contrast, TNFα stimulation significantly increased the expression of *GPx1* (*p* = 0.0286), *Catalase* (*p* = 0.0286) and *Nrf2* (*p* = 0.0286) in macrophages, however independent of radon treatment, but dependent on inflammatory stimuli, while *SOD1* expression was not significantly modulated (Figure 7J–M). Concurrently, ROS was decreased upon TNFα treatment of macrophages (Figure 7I). 

**Figure 8:**
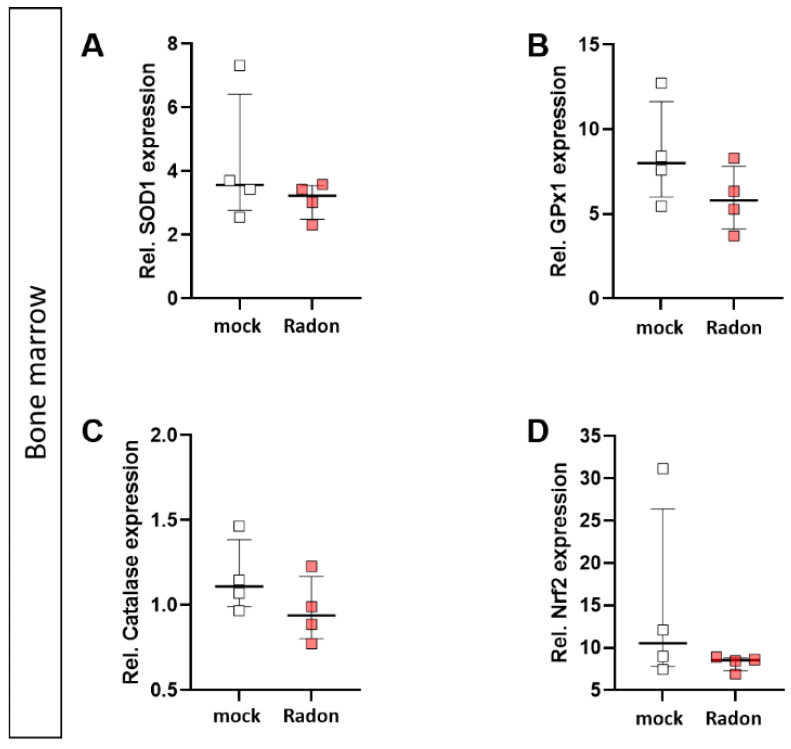
Ex vivo radon treatment of bone-marrow derived from C57Bl/6 mice has no effects on the anti-oxidative system. C57Bl/6-derived bone marrow was exposed to radon for 1 h at standard cell culture conditions. The expression of *SOD1* (**A**), *GPx1* (**B**), *Catalase* (**C**) and *Nrf2* (**D**) was measured by qPCR 24 h after radon exposure of bone marrow. 4 cell pools were exposed in 2 independent experiments. Data is presented as Median + IQR. Statistics were calculated using Mann-Whitney-U test.

In vitro exposure of C57Bl/6-derived bone marrow to radon slightly decreased expression of *GPx1* and *Catalase* (Figure 8B,C), however, these findings were not significant.

**Figure 9:**
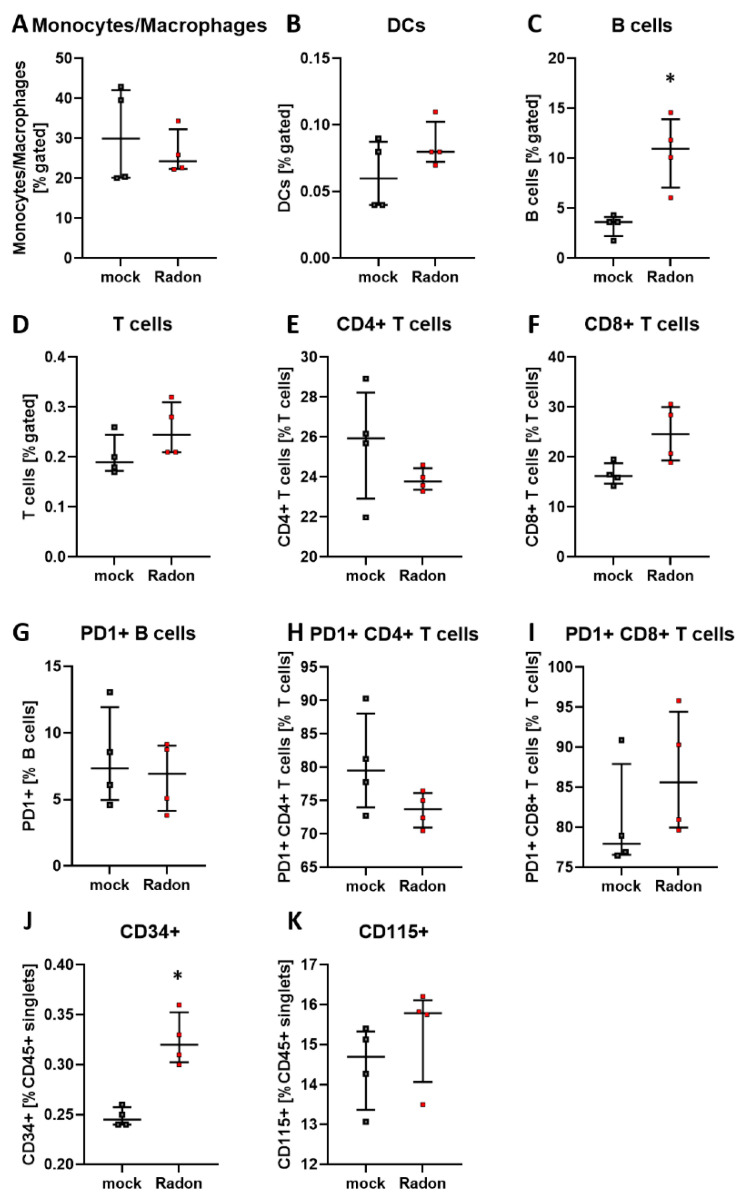
Ex vivo treatment of bone marrow cells with radon significantly increases B cell numbers and CD34+ hematopoietic precursor cells. Isolated bone marrow cells derived from C57Bl/6 mice were exposed to radon (168 kBq/m^3^) or mock treatment for one hour under standard cell culture conditions. 24 h after treatment cells were collected, stained for multicolor flow-cytometry analysis and subsequently analyzed (**A**–**K**). Four cell pools, each derived from 10 C57Bl/6 mice, were exposed in two independently performed experiments. Data is presented as Median + IQR. Statistics were calculated using Mann-Whitney-U test (* *p* < 0.05).

Similar to the peripheral blood analysis in in vivo experiments of K/BxN serum-induced mice, we found a significant increase of B cells in radon exposed bone marrow cells (Figure 9C, *p* = 0.0286). Significant alterations were also present in CD34+ hematopoietic stem cells within the radon group (Figure 9J, *p* = 0.0286), whereas only mild alterations were found in T cells as well as CD115+ monocytic precursors (Figure 9E,F,H,K).

## 4. Discussion

Similar to the observations made in patient studies, we found a significant improvement in the clinical course of RA in radon treated animals in comparison to sham irradiated control animals (Figure 1H). As radon exposure was carried out at room temperature and humidity was also not enhanced, we suggest that the observed effects regarding the clinical score (Figure 1F–H), as well as modulation of immune cells in the bone marrow (Figure 3), the peripheral blood (Figure 4) and the cytokine milieu in the serum (Figure 5) in the treatment group are radon-mediated effects. This is in line with observations made in patient studies: while probably due to the spa effects with elevated temperatures and humidity, initial improvement in treatment and control groups were observed, long lasting analgesic effects were only obvious in radon treatment groups [6,10]. This suggests radon to be responsible for inducing long-lasting effects in improvement of symptoms.

As mentioned above, ROS and the anti-oxidative system are suspected to be one of the modes of action through which radon exerts some of its molecular effects. Therefore, we examined key factors of the anti-oxidative system in the in vivo exposed animals using qPCR. However, unlike other reports [14,15,16], that indicated a significant influence of radon exposure especially on SOD1, we found no significant radon induced alterations of the anti-oxidative enzymes SOD1, GPx1, catalase, and Nrf2 in in vivo exposed animals (Figure 2). This discrepancy is most likely due to the differences in experimental set-up (dose and duration of radon treatment), time of sampling (7d vs right after exposure), differences in utilized methods, the organs that have been looked into, and the mouse model used. All the aforementioned experiments were carried out using Balb/c mice, whereas we used K/BxN serum induced C57Bl/6 mice. Further, we have shown before, that an inflammatory background is essential in the induction of anti-inflammatory effects [34,35], therefore the observed differences could also be due to the different inflammatory background. These important facts have to be well-thought-out when a radon treatment for patients is being considered. This indicates that personalized treatment approaches should be taken into mind, in dependence of the mentioned factors. Further, immune alterations might play a more important role on such individual settings. 

We performed immunephenotyping in the bone marrow (Figure 3) as well as in the peripheral blood (Figure 4) of sham or radon irradiated mice. While we expected the accumulated dose to be higher in the bone marrow [2,36], no significant alterations in immune cells besides a minor reduction in monocytes/macrophages (Figure 3A) was found. Interestingly however, in the peripheral blood, a significant increase of B cells (Figure 4B) alongside minor, non-significant alterations in T cells (Figure 4D–F,H,I) was observed. In general, B cells are considered to be positive regulators of the immune system as they produce a number of cytokines, including IL-4, -6, 10 as well as interferon (IFN)γ and TGFβ. Further, they are involved in CD4+ T cell expansion, but may also positively regulate CD8+ T cells, as it has been shown in mouse models of autoimmune diseases. Further, they can be a source of autoreactive antibodies. However, they can also be negative regulators of the immune system, especially during inflammation and autoimmunity and specific B cell subsets, so called regulatory B cells, have been reported [37,38,39,40,41]. These regulatory B cells have been identified regularly in mouse models for inflammation and autoimmunity, where several subsets of these cells have been acknowledged [39]. As we have identified B cells to be CD19+ only [33], we cannot further determine B cell subsets in our set-up, as it would be necessary to identify regulatory B cells [41,42]. Therefore, further examinations are needed in order to deeper understand the radon-mediated increase of B cell numbers and the subsequent effects. Nevertheless, one has to stress, that in the RAD-ON01 patient study, a slight temporary increase of B cells in the peripheral blood of the radon patients was observed 6 weeks after therapy [3].

Further, while we found only minor alterations in T cell numbers (Figure 4D–F), Rühle et al. suggested that radon balneology only induced minor alterations in total T cell numbers, but significant and long-lasting alterations were found in their activation status [3]. This is in line with our findings in the cytokine milieu in the peripheral blood where IL-16 (Figure 5G), IL-17A (Figure 5H) and IL-22 (Figure 5I) were slightly reduced in radon treated animals. All of these cytokines are involved in T_h_17 response [43] and could therefore point to an alteration in T cell subsets and/or activation status. Next to the slight alterations in cytokines involved in T_h_17 response, we detected a significant increase of IL-5 in the radon group (Figure 5D). IL-5 can be produced by e.g., T_h_2 cells, and there are indications that T_h_2-produced IL-5 is able to promote and expand antigen-specific Tregs that have been activated by IL-4 and autoantigens and that are involved in the suppression of autoimmunity, suggesting a beneficial effect of radon treatment in autoimmune diseases such as RA [44]. However, as our animals have been exposed to radon inhalation therapy, it is also of note that IL-5, alongside with IL-4, is also known to be involved in airway inflammation and hyperreactivity especially in asthma [45,46,47]. Next to its role in T cell biology, IL-5 is also involved in the activation of B cells as well as eosinophils, which we have found to be significantly (Figure 4B) and slightly (Figure 4C) elevated in the radon group [48,49]. 

When comparing the preclinical results with patient outcome after a radon spa treatment in Bad Steben (series of 9 baths/20 min each over a course of three weeks, natural radon spring water with 600–1200 Bq/L radon), similar alterations in the immune system were found. In that matter Rühle et al.*,* as mentioned above, observed that T cells, monocytes and eosinophils in peripheral blood were significantly increased at the first time point after therapy alongside a tendency for increased B cell numbers [3]. Similarly, in our mouse model, B cells were significantly increased in the peripheral blood while T cells, monocytes and eosinophils showed a tendency for it (Figure 4). Future research should additionally include analyses of the activation state of immune cell subsets. Again, similar to our results (Figure 5), Kullmann et al. found no significant alterations in serum levels of TNF-α, IL-10 and IL-1β [21]. With regard to the antioxidative system, Kuciel-Lewandowska et al. found, in a pilot study examining the integrated antioxidant system of the body in the course of radon therapy that the total antioxidant status of patients increased after radon therapy. However, as the applied methods differ from those we have used in our preclinical study and as patient numbers are relatively low, further research on the role of the anti-oxidative system after radon therapy is needed [17,50]. Nevertheless, of course, mice and humans cannot be compared one-to-one, and preclinical examinations can only give hints as to which parameters should be looked at in patient studies in more detail. 

Furthermore, while we chose similar exposure parameters for our animal experiments, there are, of course, other factors that need to be taken into consideration, as e.g., different anatomical composition and a higher respiratory rate in mice compared to humans. Sakoda et al. found the calculated whole lung dose to be 3-times higher in mice compared to humans [51]. However, the equilibrium factor that was used in these calculations was 0.4, suggesting comparatively high amounts of decay products. The decay products, according to the International Commission on Radiological Protection (ICRP), are responsible for most of the lung dose [52], as the radon progeny is either inhaled in a higher rate with increased respiratory rate, resulting at a higher lung dose. However, this is different in our radon chamber. Within this chamber, we have a very low amount of radon progeny (equilibrium factor ~ 0.01) and thus a homogenous distribution of radon in the air and mouse lungs, independent of respiratory frequency. Furthermore, radon has a half-life period of 3.8 days, which is a lot longer than the respiratory cycle of mice, therefore it is unlikely that radon is decaying within a breathing cycle. Thus, for pure radon, respiratory frequency is most likely not an important factor in this setting.

For the examinations in peripheral blood, we assume, according to the biokinetic models of ICRP, radon to merge directly into the blood from the lungs. Blood is able to take up radon until a saturation is reached: The blood is then transporting the dissolved radon to the organs. As mice have a higher metabolic rate than humans, this likely happens faster in mice than in humans; however, as both, mice and humans can only take up a certain amount of radon, this is independent of the amount of available radon and also independently of metabolic rates or size. Therefore, we suggest that in terms of radon uptake in the blood, our preclinical approach is comparable to the human situation. 

Further, when applying IR it is often suggested that the main effects that can be observed following exposure to IR are mainly mediated via the direct induction of cell death or ROS-induced DNA damage. However, we found no significant radon mediated alterations regarding cell death in the examined bone marrow, monocytes or macrophages (Figure 6). We then took a closer look into the induction of ROS as well as the anti-oxidative system in cells that are known to produce large amounts of ROS. Therefore, we exposed ex vivo differentiated monocytes and macrophages to radon and performed DCF-analysis as well as qPCR analysis of key factors of the anti-oxidative response system (Figure 7). No significant alterations in the induction of ROS were found, however, the DCF signal was slightly increased in monocytes after radon exposure (Figure 7C). Next to its putative effects in radon treatment, ROS is also involved in the initiation, progression, and resolution of inflammatory processes thus making it a central key player in the progression of inflammatory processes in general [53,54]. We found significant effects in healthy monocytes and macrophages only after inducing an inflammatory environment by adding TNFα (Figure 7D–G,J–M) that exceeded those of radon induced effects. We therefore suggest that the inflammatory environment causes greater alterations within the anti-oxidative system than radon. Similar to our results in in vivo exposed mice, we also found no significant alterations of anti-oxidative enzymes SOD1, GPx1, catalase, and Nrf2 in ex vivo exposed bone marrow (Figure 8). However, in line with our findings in the peripheral blood of in vivo exposed K/BxN serum-induced, we found a significant increase of B cells (Figure 9B) and here additionally a significant increase of hematopoietic precursor cells (Figure 9J) alongside slight alterations in T cells (Figure 9E,F,H,I) and a minor induction of the monocytic marker CD115 (Figure 9K). 

A possible explanation for the in part discrepant effects in in vivo versus ex vivo exposed bone marrow could be on the one hand due to the differences in solubility or radon. Sanjon et al. described that radon solubility is reduced in water (and therefore probably also cell culture medium) in comparison to fat [32]. As described by Maier et al. [2], in vivo the bone marrow holds a higher fat content as it is composed of both, bone marrow cells and fat cells. However, this was only given in our in vivo experiments as ex vivo bone marrow was flushed out and only bone marrow cells were seeded in cell culture medium. On the other hand, radon uptake differs in both models, while in in vivo radon is actively distributed in the body, in ex vivo settings radon is dissolved passively in the medium. Meaning, in vivo exposed mice inhaled the radon, which was then dissolved in blood. This way it is actively transported and thus distributed where it can reach and accumulate in the cells. On the contrary, in ex vivo treatment, radon is passively dissolved in the medium where it can only diffuse. This way, in order for α-particles deposit its energy within a cell, cells and α-particles have to be in close vicinity by chance. 

## 5. Conclusions

Taking all these results into consideration, we consequently suggest that the clinical response in arthritic mice to radon as well as the observed modulations in immune cell subsets and cytokines in the described experimental set-ups are likely not cell death- or ROS-related, but rather immune-mediated. Additionally, as in vivo radon exposed mice showed significant improvement of clinical parameters in comparison to sham irradiated animals without further co-factors such as temperature, minerals or humidity [55], a pure placebo effect can be excluded. Further, additional research is needed in order to better understand the radon-mediated effects on B cell and T cell biology. With regard to radon induced risk factors, while to date no clear answer can be given, our results also suggest that further research is needed, but that the radon-induced immune alterations are minor and therefore not actively health-threatening in this low dose and not chronic exposure scenario.

## Figures and Tables

**Table 1:** Exposition parameters for in vivo experiments within the radon chamber.

	Experiment 1		Experiment 2	
	Controls (n = 4)	Treated (n = 5)	Controls (n = 3)	Treated (n = 4)
**Temperature [°C]**	23.2 ± 0.2	23.4 ± 0.1	22.0 ± 0.1	22.3 ± 0.1
**Relative humidity [%]**	68.7 ± 1.4	71.6 ± 2.7	60.1 ± 3.2	67.1 ± 4.7
**Atmospheric pressure [mbar]**	1011 ± 3	1011 ± 3	1017 ± 3	1017 ± 3
**Radon activity concentration [kBq/m^3^]**	0.0045 ± 0.0139	466 ± 5	0.008 ± 0.0046	489.3 ± 12.5

**Table 2:** Mean exposition parameters for ex vivo experiments within the radon chamber.

	Mean Parameters for Bone Marrow Experiments		Mean Parameters for Monocytes/Macrophages Experiments	
	Controls	Treated	Controls	Treated
**Temperature [°C]**	36.15 ± 0.21	36.36 ± 0.19	36.59 ± 0.34	36.37 ± 0.12
**Relative humidity [%]**	74.35 ± 0.35	73.8 ± 0.71	77.17 ± 0.93	78 ± 0.8
**Atmospheric pressure [mbar]**	1013.5 ± 0.71	1012 ± 1.41	1007.33 ± 7.57	1007 ± 6.24
**CO_2_ [%]**	4.54 ± 0.04	4.52 ± 0.05	4,53 ± 0.14	4.47 ± 0.13
**Radon activity concentration [kBq/m^3^]**	0.098 ± 7.07	167.75 ± 11.38	0.041 ± 0.045	176.83 ± 2.99

## Data Availability

The data presented in this study are available from the corresponding author upon reasonable request.

## Appendix A

**Table A1:** Multicolor flow cytometry panels, antibodies and suppliers used in experiments.

Target	Fluorochrome	Supplier
**Myeloid Cells Panel**		
Live/Dead	Zombie NIR	BioLegend
MHC-II	e-Fluor450	eBioscience
Ly-6G	PE-Cy7	BD
Ly-6C	FITC	BD
CD11b	APC	eBioscience
Siglec-F	PE	BD
CD45.2	PerCP-Cy5.5	eBioscience
PDCA-1	Brilliant Violet 650	BioLegend
CD11c	Brilliant Violet 510	BD
**Myeloid and Precursors Panel**		
Live/dead	Zombie NIR	BioLegend
MHC-II	e-Fluor450	eBioscience
Ly-6G	PE-Cy7	BD
Ly-6C	FITC	BD
CD11b	APC	eBioscience
Siglec-F	PE	BD
CD45.2	PerCP-Cy5.5	eBioscience
CD34	Brilliant Violet 650	BD
CD115	Brilliant Violet 510	BD
**Lymphocytes Panel**		
Live Dead	Zombie Aqua	BioLegend
CD62L	PE-Cy7	BD
CD8	Brilliant Violet 605	BioLegend
FcεR1α	PE	BBioLegend
CD4	FITC	BD
CD19	APC-Cy7	BD
CD3e	V 450	BD
CD45.2	PerCP-Cy5.5	eBioscience
CD49b	APC	BioLegend
PD-1	PE-Dazzle 594	BioLegend
**Macrophage/Monocyte Panel**		
Live/Dead	Zombie NIR	BioLegend
CD11b	FITC	BD
MHC-II	eFluor450	eBioscience
F4/80	eFluor660	eBioscience
CD80	PE	BD
Ly-6C	PE-Cy7	BD
CD86	PerCP-Vio700	Miltenyi Biotech
CD206	Brilliant Violet 650	BioLegend
CD45.2	PE-Dazzle 594	BioLegend
**Checkpoint Panel**		
Live/Dead	Zombie NIR	BioLegend
PD-L1	PE-Cy7	eBioscience
PD-L2	Brilliant Violet 510	BD
HVEM/CD270	APC	BioLegend
Ox40L	PerCP-eFLuor710	eBioscience
Galectin-9	Brilliant Violet 421	BD
4-1BBL/CD137L	VioBright FITC	Miltenyi Biotech
VISTA/PD-1H	PE-Dazzle 594	BioLegend
B7-H3/CD276	Brilliant Violet 650	BD

**Table A2:** Sequences of primers and probes for quantitative real-time PCR.

Gene	Primer/Probe	Sequence (5′→3′)	Reference
***mSOD1***	**Forward**	GGACGGTGTGGCCAATGT	[56]
	**Reverse**	CGGCCAATGATGGAATGC	
	**Probe**	[FAM] TGTGATCTCACTCTCAGGAG [TAMRA]	
***mGPx1***	**Forward**	CCCCACTGCGCTCATGA	[57]
	**Reverse**	GGCACACCGGAGACCAAA	
	**Probe**	[FAM] CGACCCCAAGTACATC [TAMRA]	
***mCatalase***	**Forward**	TTCAGAAGAAAGCGGTCAAGAAT	[57]
	**Reverse**	GATGCGGGCCCCATAGTC	
	**Probe**	[FAM] CACTGACGTCCACCC [TAMRA]	
***mNfe2l2***	**Forward**	CAAGACTTGGGCCACTTAAAAGAC	[58,59]
	**Reverse**	AGTAAGGCTTTCCATCCTCATCAC	
	**Probe**	[FAM] AGGCGGCTCAGCACCTTGTATCTTGA [TAMRA]	
***mHPRT***	**Forward**	TGAAGAGCTACTGTAATGATCAGTCAAC	[60]
	**Reverse**	AGCAAGCTTGCAACCTTAACCA	
	**Probe**	[FAM] TGCTTTCCCTGGTTAAGCAGTACAGCCC [TAMRA]	

## References

[1] Gaisberger M., Fuchs J., Riedl M., Edtinger S., Reischl R., Grasmann G., Hölzl B., Landauer F., Dobias H., Eckstein F. (2021). Endogenous anandamide and self-reported pain are significantly reduced after a 2-week multimodal treatment with and without radon therapy in patients with knee osteoarthritis: A pilot study. Int. J. Biometeorol..

[2] Maier A., Wiedemann J., Rapp F., Papenfuß F., Rödel F., Hehlgans S., Gaipl U.S., Kraft G., Fournier C., Frey B. (2021). Radon Exposure—Therapeutic Effect and Cancer Risk. Int. J. Mol. Sci..

[3] Rühle P.F., Wunderlich R., Deloch L., Fournier C., Maier A., Klein G., Fietkau R., Gaipl U.S., Frey B. (2017). Modulation of the peripheral immune system after low-dose radon spa therapy: Detailed longitudinal immune monitoring of patients within the RAD-ON01 study. Autoimmunity.

[4] Paz N., Hartel C., Nasonova E., Donaubauer A.J., Frey B., Ritter S. (2021). Chromosome Aberrations in Lymphocytes of Patients Undergoing Radon Spa Therapy: An Explorative mFISH Study. Int. J. Environ. Res. Public Health.

[5] Rühle P.F., Klein G., Rung T., Tiep Phan H., Fournier C., Fietkau R., Gaipl U.S., Frey B. (2019). Impact of radon and combinatory radon/carbon dioxide spa on pain and hypertension: Results from the explorative RAD-ON01 study. Mod. Rheumatol..

[6] Franke A., Reiner L., Pratzel H.G., Franke T., Resch K.L. (2000). Long-term efficacy of radon spa therapy in rheumatoid arthritis—A randomized, sham-controlled study and follow-up. Rheumatology.

[7] Franke A., Reiner L., Resch K.L. (2007). Long-term benefit of radon spa therapy in the rehabilitation of rheumatoid arthritis: A randomised, double-blinded trial. Rheumatol. Int..

[8] Herold M., Lind-Albrecht G. (2008). Radon within therapeutic strategies of ankylosing spondylitis. Wien. Med. Wochenschr..

[9] Rühle A., Tkotsch E., Mravlag R., Haehl E., Spohn S.K.B., Zamboglou C., Huber P.E., Debus J., Grosu A.-L., Sprave T. (2021). Low-dose radiotherapy for painful osteoarthritis of the elderly: A multicenter analysis of 970 patients with 1185 treated sites. Strahlenther. Onkol..

[10] Falkenbach A., Kovacs J., Franke A., Jörgens K., Ammer K. (2005). Radon therapy for the treatment of rheumatic diseases—Review and meta-analysis of controlled clinical trials. Rheumatol. Int..

[11] Van Tubergen A., Boonen A., Landewé R., Rutten-Van Mölken M., Van Der Heijde D., Hidding A., Van Der Linden S. (2002). Cost effectiveness of combined spa-exercise therapy in ankylosing spondylitis: A randomized controlled trial. Arthritis Rheum..

[12] van Tubergen A., Landewé R., van der Heijde D., Hidding A., Wolter N., Asscher M., Falkenbach A., Genth E., Thè H.G., van der Linden S. (2001). Combined spa-exercise therapy is effective in patients with ankylosing spondylitis: A randomized controlled trial. Arthritis Rheum..

[13] Kataoka T. (2013). Study of antioxidative effects and anti-inflammatory effects in mice due to low-dose X-irradiation or radon inhalation. J. Radiat. Res..

[14] Kataoka T., Kanzaki N., Sakoda A., Shuto H., Yano J., Naoe S., Tanaka H., Hanamoto K., Terato H., Mitsunobu F. (2021). Evaluation of the redox state in mouse organs following radon inhalation. J. Radiat. Res..

[15] Kataoka T., Shuto H., Naoe S., Yano J., Kanzaki N., Sakoda A., Tanaka H., Hanamoto K., Mitsunobu F., Terato H. (2021). Radon inhalation decreases DNA damage induced by oxidative stress in mouse organs via the activation of antioxidative functions. J. Radiat. Res..

[16] Kobashi Y., Kataoka T., Kanzaki N., Ishida T., Sakoda A., Tanaka H., Ishimori Y., Mitsunobu F., Yamaoka K. (2020). Comparison of antioxidative effects between radon and thoron inhalation in mouse organs. Radiat. Environ. Biophys..

[17] Kuciel-Lewandowska J., Gnus J., Pawlik-Sobecka L., Płaczkowska S., Kokot I., Kasperczak M., Paprocka-Borowicz M. (2018). The Assessment of the Integrated Antioxidant System of the Body in the Course of Radon Therapy: A Pilot Study. Biomed. Res. Int..

[18] Nie J.H., Chen Z.H., Liu X., Wu Y.W., Li J.X., Cao Y., Hei T.K., Tong J. (2012). Oxidative damage in various tissues of rats exposed to radon. J. Toxicol. Environ. Health A.

[19] Nagarkatti M., Nagarkatti P.S., Brooks A. (1996). Effect of radon on the immune system: Alterations in the cellularity and functions of T cells in lymphoid organs of mouse. J. Toxicol. Environ. Health.

[20] Cucu A., Shreder K., Kraft D., Rühle P.F., Klein G., Thiel G., Frey B., Gaipl U.S., Fournier C. (2017). Decrease of Markers Related to Bone Erosion in Serum of Patients with Musculoskeletal Disorders after Serial Low-Dose Radon Spa Therapy. Front. Immunol..

[21] Kullmann M., Rühle P.F., Harrer A., Donaubauer A., Becker I., Sieber R., Klein G., Fournier C., Fietkau R., Gaipl U.S. (2019). Temporarily increased TGFβ following radon spa correlates with reduced pain while serum IL-18 is a general predictive marker for pain sensitivity. Radiat. Environ. Biophys..

[22] Arenas M., Algara M., De Febrer G., Rubio C., Sanz X., de la Casa M.A., Vasco C., Marín J., Fernández-Letón P., Villar J. (2021). Could pulmonary low-dose radiation therapy be an alternative treatment for patients with COVID-19 pneumonia? Preliminary results of a multicenter SEOR-GICOR nonrandomized prospective trial (IPACOVID trial). Strahlenther. Onkol..

[23] Droge W. (2002). Free radicals in the physiological control of cell function. Physiol. Rev..

[24] Ray P.D., Huang B.W., Tsuji Y. (2012). Reactive oxygen species (ROS) homeostasis and redox regulation in cellular signaling. Cell. Signal..

[25] Kaspar J.W., Niture S.K., Jaiswal A.K. (2009). Nrf2:INrf2 (Keap1) signaling in oxidative stress. Free Radic. Biol. Med..

[26] Craige S.M., Kant S., Keaney J.F. (2015). Reactive oxygen species in endothelial function—From disease to adaptation. Circ. J..

[27] Maier A., van Beek P., Hellmund J., Durante M., Schardt D., Kraft G., Fournier C. (2015). Experimental setup for radon exposure and first diffusion studies using gamma spectroscopy. Nucl. Instrum. Meth. B.

[28] Mititelu R.R., Pădureanu R., Băcănoiu M., Pădureanu V., Docea A.O., Calina D., Barbulescu A.L., Buga A.M. (2020). Inflammatory and Oxidative Stress Markers—Mirror Tools in Rheumatoid Arthritis. Biomedicines.

[29] Christensen A.D., Haase C., Cook A.D., Hamilton J.A. (2016). K/BxN Serum-Transfer Arthritis as a Model for Human Inflammatory Arthritis. Front. Immunol..

[30] Kouskoff V., Korganow A.S., Duchatelle V., Degott C., Benoist C., Mathis D. (1996). Organ-specific disease provoked by systemic autoimmunity. Cell.

[31] Korn M.A., Schmitt H., Angermüller S., Chambers D., Seeling M., Lux U.T., Brey S., Royzman D., Brückner C., Popp V. (2020). Siglec-15 on Osteoclasts Is Crucial for Bone Erosion in Serum-Transfer Arthritis. J. Immunol..

[32] Sanjon E.P., Maier A., Hinrichs A., Kraft G., Drossel B., Fournier C. (2019). A combined experimental and theoretical study of radon solubility in fat and water. Sci. Rep..

[33] Rückert M., Deloch L., Frey B., Schlücker E., Fietkau R., Gaipl U.S. (2021). Combinations of Radiotherapy with Vaccination and Immune Checkpoint Inhibition Differently Affect Primary and Abscopal Tumor Growth and the Tumor Microenvironment. Cancers.

[34] Deloch L., Rückert M., Fietkau R., Frey B., Gaipl U.S. (2018). Low-Dose Radiotherapy Has No Harmful Effects on Key Cells of Healthy Non-Inflamed Joints. Int. J. Mol. Sci..

[35] Deloch L., Derer A., Hueber A.J., Herrmann M., Schett G.A., Wölfelschneider J., Hahn J., Rühle P.F., Stillkrieg W., Fuchs J. (2018). Low-Dose Radiotherapy Ameliorates Advanced Arthritis in hTNF-α tg Mice by Particularly Positively Impacting on Bone Metabolism. Front. Immunol..

[36] Henshaw D.L., Eatough J.P., Richardson R.B. (1990). Radon as a causative factor in induction of myeloid leukaemia and other cancers. Lancet.

[37] Bouaziz J.D., Yanaba K., Tedder T.F. (2008). Regulatory B cells as inhibitors of immune responses and inflammation. Immunol. Rev..

[38] Fetter T., Niebel D., Braegelmann C., Wenzel J. (2020). Skin-Associated B Cells in the Pathogenesis of Cutaneous Autoimmune Diseases-Implications for Therapeutic Approaches. Cells.

[39] Miyagaki T., Fujimoto M., Sato S. (2015). Regulatory B cells in human inflammatory and autoimmune diseases: From mouse models to clinical research. Int. Immunol..

[40] Mauri C. (2010). Regulation of immunity and autoimmunity by B cells. Curr. Opin. Immunol..

[41] Mizoguchi A., Bhan A.K. (2006). A case for regulatory B cells. J. Immunol..

[42] DiLillo D.J., Matsushita T., Tedder T.F. (2010). B10 cells and regulatory B cells balance immune responses during inflammation, autoimmunity, and cancer. Ann. N. Y. Acad. Sci..

[43] Wilson N.J., Boniface K., Chan J.R., McKenzie B.S., Blumenschein W.M., Mattson J.D., Basham B., Smith K., Chen T., Morel F. (2007). Development, cytokine profile and function of human interleukin 17-producing helper T cells. Nat. Immunol..

[44] Tran G.T., Hodgkinson S.J., Carter N.M., Verma N.D., Plain K.M., Boyd R., Robinson C.M., Nomura M., Killingsworth M., Hall B.M. (2012). IL-5 promotes induction of antigen-specific CD4+CD25+ T regulatory cells that suppress autoimmunity. Blood.

[45] Hogan S.P., Koskinen A., Matthaei K.I., Young I.G., Foster P.S. (1998). Interleukin-5-producing CD4+ T cells play a pivotal role in aeroallergen-induced eosinophilia, bronchial hyperreactivity, and lung damage in mice. Am. J. Respir. Crit. Care Med..

[46] Hogan S.P., Matthaei K.I., Young J.M., Koskinen A., Young I.G., Foster P.S. (1998). A novel T cell-regulated mechanism modulating allergen-induced airways hyperreactivity in BALB/c mice independently of IL-4 and IL-5. J. Immunol..

[47] Hogan S.P., Mould A., Kikutani H., Ramsay A.J., Foster P.S. (1997). Aeroallergen-induced eosinophilic inflammation, lung damage, and airways hyperreactivity in mice can occur independently of IL-4 and allergen-specific immunoglobulins. J. Clin. Investig..

[48] Takatsu K., Moon B.-G., Itakura A., Tsukamoto Y., Horikawa K., Ikutani M., Kouro T., Takaki S. (2005). Role of IL-5 in the innate immune system and disease control. Int. Congr. Ser..

[49] Harriman G.R., Kunimoto D.Y., Elliott J.F., Paetkau V., Strober W. (1988). The role of IL-5 in IgA B cell differentiation. J. Immunol..

[50] Kuciel-Lewandowska J.M., Pawlik-Sobecka L., Płaczkowska S., Kokot I., Paprocka-Borowicz M. (2018). The assessment of the integrated antioxidant system of the body and the phenomenon of spa reaction in the course of radon therapy: A pilot study. Adv. Clin. Exp. Med..

[51] Sakoda A., Ishimori Y., Fukao K., Yamaoka K., Kataoka T., Mitsunobu F. (2012). Lung dosimetry of inhaled radon progeny in mice. Radiat. Environ. Biophys..

[52] ICRP (2017). Occupational intakes of radionuclides: Part 3. ICRP Publication 137. Ann. ICRP.

[53] Chelombitko M.A. (2018). Role of Reactive Oxygen Species in Inflammation: A Minireview. Mosc. Univ. Biol. Sci. Bull..

[54] Mittal M., Siddiqui M.R., Tran K., Reddy S.P., Malik A.B. (2014). Reactive oxygen species in inflammation and tissue injury. Antioxid. Redox Signal..

[55] Karagülle M., Karagülle M.Z. (2015). Effectiveness of balneotherapy and spa therapy for the treatment of chronic low back pain: A review on latest evidence. Clin. Rheumatol..

[56] Lewis P., Stefanovic N., Pete J., Calkin A.C., Giunti S., Thallas-Bonke V., Jandeleit-Dahm K.A., Allen T.J., Kola I., Cooper M.E. (2007). Lack of the antioxidant enzyme glutathione peroxidase-1 accelerates atherosclerosis in diabetic apolipoprotein E-deficient mice. Circulation.

[57] de Haan J.B., Witting P.K., Stefanovic N., Pete J., Daskalakis M., Kola I., Stocker R., Smolich J.J. (2006). Lack of the antioxidant glutathione peroxidase-1 does not increase atherosclerosis in C57BL/J6 mice fed a high-fat diet. J. Lipid Res..

[58] Honkura Y., Matsuo H., Murakami S., Sakiyama M., Mizutari K., Shiotani A., Yamamoto M., Morita I., Shinomiya N., Kawase T. (2016). NRF2 Is a Key Target for Prevention of Noise-Induced Hearing Loss by Reducing Oxidative Damage of Cochlea. Sci. Rep..

[59] Tsujita T., Peirce V., Baird L., Matsuyama Y., Takaku M., Walsh S.V., Griffin J.L., Uruno A., Yamamoto M., Hayes J.D. (2014). Transcription factor Nrf1 negatively regulates the cystine/glutamate transporter and lipid-metabolizing enzymes. Mol. Cell. Biol..

[60] Hori S., Nomura T., Sakaguchi S. (2003). Control of regulatory T cell development by the transcription factor Foxp3. Science.

